# Interaction between physical activity and sleep duration in relation to insulin resistance among non-diabetic Chinese adults

**DOI:** 10.1186/1471-2458-12-247

**Published:** 2012-03-28

**Authors:** Hui Zuo, Zumin Shi, Baojun Yuan, Yue Dai, Gang Hu, Gaolin Wu, Akhtar Hussain

**Affiliations:** 1 Department of Nutrition and Food Hygiene, Jiangsu Provincial Center for Disease Control and Prevention, 172 Jiangsu Road, Nanjing 210009, China; 2 Department of General Practice and Community Medicine, Institute of Health and Society, Faculty of Medicine, University of Oslo, Oslo, Norway; 3 Discipline of Medicine, University of Adelaide, Adelaide, Australia; 4 Pennington Biomedical Research Center, Baton Rouge, Louisiana, USA

**Keywords:** Domestic, Occupational, Transportation, Leisure, Physical activity, Sleep duration, Insulin resistance

## Abstract

**Background:**

It is of a public health interest to explore the relationship between different types of physical activity, sleep duration and diabetes/insulin resistance. However, little is known about such relationship. This study examines the single and joint associations of different types of physical activity, and sleep duration on insulin resistance among non-diabetic Chinese adults.

**Methods:**

Data was collected from 1124 non-diabetic adults in Jiangsu Province from the China Health and Nutrition Survey (CHNS). Domestic, occupational, transportation and leisure physical activity were assessed in terms of metabolic equivalent (MET)-hours-per-week to account for both intensity and time spent. Sleep duration was categorized into three groups: ≤ 7 hours, 7-9 hours, and ≥ 9 hours. Insulin resistance was evaluated by the homeostasis model of assessment (HOMA) and defined as the highest quartile of HOMA.

**Results:**

Total physical activity was mainly composed of occupational activity (75.1%), followed orderly by domestic, transportation and leisure time activity in both men and women. Total physical activity level was strongly negatively associated with fasting insulin and HOMA (*p *< 0.001). Among four types of physical activity, occupational activity was significantly negatively associated with HOMA after full adjustment (*p *< 0.05). Transportation activity was also negatively associated with HOMA when adjusted for age and gender (*p *= 0.028). Moreover, the combination of low physical activity and short sleep duration was associated with the highest odds of insulin resistance (adjusted OR = 3.26, 95% CI: 1.57-6.78), compared to those with high physical activity and adequate sleep duration.

**Conclusions:**

Physical activity, mainly occupational physical activity, was negatively associated with insulin resistance in non-diabetic Chinese population, independently of potential confounders. There was a synergic effect of low physical activity and short sleep duration on insulin resistance.

## Background

Physical activity is now recognized as a key determinant in the promotion and maintenance of human health. Physical inactivity is one of the leading risk factors of major non-communicable diseases such as type 2 diabetes and cardiovascular diseases (CVD) [1], whereas insulin resistance is linked closely to the development of such diseases [2,3].

Physical activity can reduce insulin resistance [4-7]. It is of a public health interest to explore the relationship between different types of physical activity and diabetes/insulin resistance. However, such information has been rarely studied. Hu *et al. *found that moderate/high occupational, commuting and leisure time activity could reduce the risk of type 2 diabetes [8] and reduce total and CVD mortality among patients with type 2 diabetes [9]. An Iranian study also indicated an inverse association between physical activity (including work, commuting and recreation) and insulin resistance [10]. No study can be found in this field in Chinese population.

Epidemiological studies have shown that short sleep duration may be related to cardiovascular disease [11-13], hypertension [13], adiposity [14,15], impaired glucose tolerance, and type 2 diabetes [16-19]. Similarly, redundant sleep duration may also be associated with higher risk of such chronic disorders [20,21]. Sleep and physical activity are interrelated. For example, physical activity may improve sleep quality [22], while sleep restriction may reduce physical activity [18]. However, they may have completely different physiological mechanisms in relation to human health. Little is known on the joint effect of sleep duration and physical activity on insulin resistance.

Non-communicable diseases like type 2 diabetes and metabolic syndrome have been steadily increasing, creating an important public health challenge in China [23-25]. This is mainly due to rapid urbanization, westernized lifestyle and the "nutrition transition" [26].

The purpose of this study was twofold: 1) to assess the association between four types of physical activity (domestic, occupational, transportation and leisure physical activity) and insulin resistance; and 2) to assess the combined effect of physical activity and sleep duration on insulin resistance in non-diabetic Chinese adults.

## Methods

### Study population

The China Health and Nutrition Survey (CHNS) is an ongoing longitudinal survey in nine diverse provinces in China, which was designed to examine the association between the economic transformation and health and nutrition status of Chinese population [27,28]. Jiangsu was the only province that collected fasting blood samples in the CHNS for the first time in 2006. This paper used the cross-sectional data collected from the 2006 CHNS in Jiangsu Province. The study sample was drawn from six areas (Suzhou, Yangzhou, Shuyang, Taixing, Haimen, and Jinhu) by a multistage random cluster process. In total, 16 villages and townships within the counties and 8 urban and suburban neighborhoods within the cities were selected randomly. Participants who had previously or newly diagnosed diabetes were excluded. Data for 1124 non-diabetic adults (504 men and 620 women) aged 18-89 years were used in this analysis. All participants provided written informed consent. The study protocol was approved by the review board in Jiangsu Provincial Center for Disease Control and Prevention. English version of the questionnaire for adults which was used for the 2006 CHNS can be found at the CHNS website [29].

### Physical activity assessment

This was collected by staff-administered questionnaires. Related questions about physical activity have been evaluated previously [30]. Four categories were included: domestic, occupational, transportation and leisure physical activity. Assessment was in terms of metabolic equivalent (MET)-hours-per-week to account for both intensity and time spent on activities [28]. All physical activities were reported in average hours-per-week spent in the past year. The level of physical activity was the product of time spent in each activity multiplied by specific MET values based on the "Compendium of Physical Activities" [31].

Measurement of domestic activity was based on four activities. The MET values assigned were as follows: 2.3 for buying food, 2.25 for preparing food or cooking, 2.15 for laundry, and 3.0 for sweeping rooms. Occupational activity was measured according to the intensity of specific jobs (light, moderate and heavy), with assigned values of 2.0, 4.0 and 6.0 respectively. Measurement of transportation activity was based on four commuting types to and from work or school. The MET values assigned were as follows: 3.0 for walking, 4.0 for bicycling, 1.5 for motorized vehicle. The MET values used for leisure activities were: 4.5 for martial arts, 5.0 for gymnastics, dancing or acrobatics, 7.5 for jogging or swimming, 6.0 for playing soccer, basketball or tennis, 5.0 for playing badminton, volleyball or pingpong.

### Sleep duration

The number of hours of sleep were assessed with the staff-administered questionnaire [29] by the question 'How much time each day do you usually spend in bed either sleeping or lying there, including nighttime (hours)?' Since no established classification criteria for sleep duration can be found from published data, we then categorized the variable into three groups: ≤ 7 hours, 7-9 hours, and ≥ 9 hours mainly based on the data distribution and sleep reality among Chinese population [32].

### Anthropometric data

Height and weight were measured directly by trained health workers who followed standard protocols. Weight in light clothing and without shoes was measured to the nearest 0.1 kg and height was measured to the nearest 0.1 cm. Waist circumference was measured at the midpoint between the lower rib margin and the iliac crest, and hip circumference at the level of maximum posterior extension of the buttocks with a non-stretch tape which was calibrated weekly. Waist and hip circumferences were recorded to the nearest 0.1 cm. Waist-hip ratio (WHR) were used as an indicator of adiposity.

### Dietary assessment

A validated semi-quantitative food frequency questionnaire (FFQ) was used to collect dietary intake information [33]. Participants were asked to recall their usual frequency and quantity of intakes of 33 food groups and beverages during the previous year with a series of detailed questions. Intakes of total energy and fat were computed by using the Chinese Food Composition Table [14]. Total energy and fat intakes which could mediate the association between physical activity and insulin resistance were adjusted in the models.

### Laboratory measurements

Venous blood samples were obtained in the morning after overnight fasting. The fasting status was verbally confirmed by subjects before the blood sampling. All blood samples were collected in three vacuum tubes and processed within three hours. The serum was stored at -70°C until laboratory assays. Fasting glucose was assessed by an enzymology method using OLYMPUS Chemistry Analyzer AU400 (Mishima Olympus CO., LTD, Shizuoka-ken, Japan). High-sensitivity C-reactive protein (hs-CRP) was determined by immunoturbidimetry assay (DiaSys Diagnostic Systems GmbH, Holzheim, Germany). Plasma insulin was measured by ELISA Kit (Millipore Corporation, Billerica, MA, USA). The homeostasis model of assessment (HOMA), calculated as fasting insulin (mU/L) * fasting glucose (mmol/L)/22.5 [34], and its highest quartile was used to evaluate insulin resistance. The presence of diabetes was based on a fasting plasma glucose level of ≥ 7.0 mmol/L according to the latest American Diabetes Association guidelines [35], as well as an existing physician's diagnosis of diabetes, with the exception of gestational diabetes.

### Additional variables

Information about age, gender, education, etc. was obtained by the questionnaire. Current smoking status (yes/no) and alcohol consumption in the past year (yes/no) were coded as dichotomous variables. The socio-demographic variables included place of residence (urban/rural), family income and education. Coding of education was based on how many years of formal education a person had completed at each level. High school included technical or vocational training at a technical school. College also included university.

### Statistical analysis

Participants' characteristics were presented as mean values with standard deviations for continuous variables, and percentages for categorical variables. The distributions of fasting insulin, HOMA and hs-CRP were skewed to the right and therefore normalized by natural logarithmic transformation (ln). Geometric means were reported for these variables. Due to familial clustering of subjects, we used generalized linear mixed-effects models (PROC MIXED in SAS, SAS Institute Inc, Cary, NC) to compute adjusted means for HOMA across tertiles of different types of physical activity.

Association between different types of physical activity and insulin resistance were evaluated by multivariate logistic regression models. Potential confounders such as age, sex, WHR, education, residence, income, smoking, alcohol drinking, dietary intakes, physical activities and hs-CRP were controlled for in the analysis. These variables were chosen according to previous publications and theoretical considerations. Medians for tertiles of each type of physical activity were used to test the linear trend in logistic regression analyses. Additionally, the combined effect of physical activity and sleep duration on insulin resistance was assessed.

A p-value of < 0.05 was considered to indicate statistical significance. All statistical analyses were conducted using the Statistical Analysis System (version 8.1, SAS Institute Inc, Cary, NC).

## Results

### Sample characteristics

Of the 1124 non-diabetic participants, 44.8% were men. The mean age was 49.2 ± 14.4 years in men and 47.9 ± 14.1 years in women. Total physical activity was mainly composed of occupation activity, followed orderly by domestic, transportation and leisure time activity both in men and women (as a whole, the constituent ratio for occupation, domestic, transportation and leisure time activity was 75.1%, 18.3%, 4.1% and 2.5% respectively). Table 1 shows the characteristics of the sample in this study according to the quartiles of HOMA. Total physical activity level, together with occupational activity was strongly negatively associated with HOMA (*p *< 0.001). Generally, a higher level of HOMA was associated with younger age, higher education, urban residence, higher income, less smoking and drinking, and low dietary energy intake. WHR, fasting glucose and fasting insulin were positively associated with HOMA (*p *< 0.001). Furthermore, an inverse association between physical activity level and fasting insulin was found (*p *< 0.001, data not shown).

**Table 1:** Baseline characteristics of 1124 adults aged 18-89 according to quartiles of HOMA in Jiangsu from the China Health and Nutrition Survey (CHNS)

*Characteristics*	*Quartiles of HOMA^a^*				
	*Q1^b^*	*Q2*	*Q3*	*Q4*	*p value for trend^c^*
HOMA (units)					
*Median*	0.45	0.85	1.33	2.33	
*Range*	0.13-0.66	0.66-1.08	1.08-1.67	1.67-14.2	
*N*	281	281	281	281	
Total physical activity (MET-hrs/week)^d^	185.1 (122.6)	167.6 (121.4)	160.7 (114.1)	144.7 (101.0)	< 0.001
*Domestic activity*	26.0 (24.7)	30.0 (25.5)	32.9 (25.9)	31.5 (28.1)	0.011
*Occupational activity*	148.8 (124.7)	126.4 (122.4)	117.3 (117.7)	102.0 (103.7)	< 0.001
*Transportation activity*	7.6 (11.0)	7.1 (12.2)	6.3 (8.1)	5.7 (7.5)	0.121
*Leisure time activity*	2.7 (13.6)	4.0 (14.6)	4.2 (13.7)	5.5 (16.9)	0.173
Age (years)	50.9 (14.0)	49.3 (13.7)	46.8 (14.3)	46.9 (14.7)	0.001
Male (%)	60.9	43.4	36.7	38.4	< 0.001
Education (%)					
*Primary school*	42.7	45.2	43.9	35.6	0.021
*High school*	53.4	49.8	48.9	56.6	
*College*	3.9	5.0	7.1	7.8	
Urban residents (%)	22.1	31.0	36.7	35.6	< 0.001
Income (Yuan, per capita)					
*Low (< 5000)*	42.9	36.4	35.0	28.9	0.005
*Medium (5000-10000)*	33.5	35.2	33.6	40.9	
*High (> 10000)*	23.7	28.5	31.4	30.2	
Current smokers (%)	36.8	27.4	18.5	15.7	< 0.001
Alcohol drinking (%)	47.0	31.7	29.5	25.3	< 0.001
Dietary intake					
*Total energy (kcal/d)*	2603.0 (1140.3)	2464.6 (888.3)	2386.7 (1149.8)	2344.8 (804.0)	0.025
*Fat (g/d)*	63.7 (26.9)	69.5 (32.3)	64.3 (32.7)	66.6 (30.5)	0.142
WHR	0.85 (0.09)	0.85 (0.08)	0.86 (0.07)	0.88 (0.07)	< 0.001
Fasting glucose (mmol/L)	4.8 (0.6)	4.9 (0.5)	5.0 (0.6)	5.3 (0.7)	< 0.001
Fasting insulin (uU/ml)	2.0 (0.9)	4.0 (0.7)	6.1 (1.1)	11.7 (6.2)	< 0.001
hs-CRP (mmol/L)	1.4 (3.3)	1.3 (2.6)	1.3 (3.0)	1.8 (3.1)	0.159
Sleep duration (hours)	8.3 (1.1)	8.3 (1.2)	8.2 (1.1)	8.2 (1.2)	0.751

^a^Numbers represent means (SD) unless otherwise indicated.

^b^Q, quartile;

^c^P-value for linear trend for continuous variables (general linear model, PROC MIXED in SAS) and for categorical variables (*χ*2 test for trend)

^d^MET, metabolic equivalent

### Single association between physical activity, sleep duration and insulin resistance

In order to further examine the association between different types of physical activity, sleep duration and insulin resistance, the mean of HOMA across different levels of physical activity and sleep duration together with the linear trend was calculated and the result is presented in Table 2. Since the overwhelming majority of the study population had a score of 0 for leisure physical activity, which was regarded as reference, the remaining sample was categorized into two groups by its median.

**Table 2:** Mean ± SE of HOMA^a ^across different levels of domestic, occupational, transportation, leisure physical activities and sleep duration in Jiangsu CHNS study

	*N*	*Crude*	*Model 1^b^*	*Model 2^c^*	*Model 3^d^*
**Domestic**					
T1^e^	374	0.95 ± 0.04	1.04 ± 0.04	1.09 ± 0.05	1.09 ± 0.05
T2	375	0.99 ± 0.04	0.98 ± 0.04	0.98 ± 0.04	0.98 ± 0.04
T3	375	1.11 ± 0.04	1.02 ± 0.04	0.99 ± 0.05	0.99 ± 0.05
p for trend		0.013	0.652	0.281	0.262
**Occupational**					
T1	370	1.11 ± 0.04	1.15 ± 0.04	1.12 ± 0.05	1.12 ± 0.05
T2	370	1.07 ± 0.04	1.03 ± 0.04	1.04 ± 0.04	1.04 ± 0.04
T3	384	0.88 ± 0.04	0.88 ± 0.04	0.92 ± 0.04	0.92 ± 0.04
p for trend		< 0.001	< 0.001	0.024	0.020
**Transportation**					
T1	374	1.10 ± 0.04	1.14 ± 0.04	1.05 ± 0.05	1.05 ± 0.05
T2	313	0.98 ± 0.04	0.96 ± 0.04	1.00 ± 0.05	1.00 ± 0.05
T3	437	0.96 ± 0.04	0.95 ± 0.04	1.00 ± 0.04	1.01 ± 0.04
p for trend		0.037	0.003	0.754	0.807
**Leisure time**					
0	1008	0.99 ± 0.02	0.98 ± 0.02	1.01 ± 0.03	1.01 ± 0.03
< Median	54	1.25 ± 0.10	1.28 ± 0.10	1.09 ± 0.11	1.10 ± 0.12
≥ Median	62	1.26 ± 0.10	1.34 ± 0.10	1.14 ± 0.11	1.13 ± 0.11
p for trend		0.006	< 0.001	0.478	0.524
**Sleep duration**					
7-9 h	510	1.01 ± 0.03	1.01 ± 0.03	1.00 ± 0.04	1.00 ± 0.04
≥ 9 h	374	0.95 ± 0.04	0.94 ± 0.04	0.99 ± 0.04	0.99 ± 0.04
≤ 7 h	240	1.12 ± 0.05	1.14 ± 0.05	1.11 ± 0.05	1.11 ± 0.05
p for trend		0.028	0.010	0.178	0.202

^a^HOMA data were expressed as geometric mean; SE, standard error.

^b^Model 1: Adjusted for age and sex using generalized linear models (PROC MIXED in SAS; SAS Institute, Cary, NC);

^c^Model 2: Adjusted for variables in model 1 plus WHR, education (low/medium/high), urban (yes/no), income (low/medium/high), smoking (yes/no), alcohol drinking (yes/no), dietary intakes, other types of physical activity (total physical activity for sleep duration);

^d^Model 3: Adjusted for variables in model 2 plus hs-CRP.

^e^T, tertile.

A significant decreasing trend of HOMA was observed across increasing tertiles of occupational physical activity in the crude and multivariable adjusted analysis (*p *< 0.05). Transportation activity was also identified to be negatively associated with HOMA both in the crude analysis and when adjusted for age and sex. But such an association became non-significant after further adjustment for other variables. Unexpectedly, leisure time activity was found to be positively associated with HOMA both in the crude analysis and when adjusted for age, sex. Similar to transportation activity, the association did not persist after further adjustment. Sleep duration was also positively associated with HOMA both in the crude analysis and when adjusted for age and sex. There was no statistical significance between them after further adjustment although the trend existed.

Table 3 presents the results of logistic regression analysis for the association between the four types of physical activity, sleep duration and insulin resistance. Occupational physical activity was negatively associated with insulin resistance among non-diabetic Chinese adults in the multivariable analyses (*p *< 0.05). Compared to the lowest tertile, subjects in the highest tertile of occupational physical activity could reduce the odds for insulin resistance by 39% in the population. Transportation activity was also negatively associated with HOMA when only adjusted for age and gender (*p *= 0.028), whereas leisure physical activity was positively associated with HOMA when adjusted for age and gender (*p *= 0.013). Such significance disappeared after further adjustment for potential confounders. There was no statistical significance between sleep duration and insulin resistance although a trend was observed.

**Table 3:** Odds ratios and 95% confidence intervals for insulin resistance according to different types of physical activity and sleep duration among non-diabetic Chinese adults in Jiangsu Province, China

	*Crude*	*Model 1^a^*	*Model 2^b^*	*Model 3^c^*
**Domestic**				
T1^d^	1.00	1.00	1.00	1.00
T2	0.79(0.54-1.15)	0.65(0.43-1.00)	0.64(0.41-1.01)	0.64(0.41-1.00)
T3	1.05(0.73-1.51)	0.74(0.47-1.18)	0.72(0.44-1.17)	0.71(0.44-1.16)
p value for trend	0.739	0.303	0.264	0.254
**Occupational**				
T1	1.00	1.00	1.00	1.00
T2	0.96(0.67-1.37)	0.78(0.53-1.15)	0.93(0.51-1.68)	0.92(0.51-1.67)
T3	0.58(0.40-0.85)	0.49(0.32-0.73)	0.63(0.34-1.16)	0.61(0.33-1.13)
p value for trend	0.003	< 0.001	0.037	0.031
**Transportation**				
T1	1.00	1.00	1.00	1.00
T2	0.83(0.57-1.21)	0.69(0.46-1.04)	0.95(0.52-1.73)	0.97(0.53-1.77)
T3	0.74(0.52-1.06)	0.62(0.42-0.91)	0.91(0.51-1.65)	0.92(0.51-1.67)
p value for trend	0.118	0.028	0.958	0.979
**Leisure time**				
0	1.00	1.00	1.00	1.00
< Median	1.35(0.68-2.71)	1.42(0.70-2.87)	1.02(0.48-2.15)	1.03(0.49-2.18)
≥ Median	1.72(0.95-3.13)	2.08(1.13-3.85)	1.23(0.63-2.43)	1.19(0.60-2.36)
p value for trend	0.056	0.013	0.549	0.618
**Sleep duration**				
7-9 h	1.00	1.00	1.00	1.00
≥ 9 h	1.14(0.81-1.61)	1.16(0.82-1.64)	1.25(0.87-1.81)	1.25(0.87-1.80)
≤ 7 h	1.27(0.86-1.87)	1.32(0.89-1.95)	1.23(0.81-1.85)	1.21(0.80-1.83)
p value for trend	0.216	0.157	0.252	0.277

^a^Model 1: Adjusted for age and sex;

^b^Model 2: Further adjusted for WHR, education (low/medium/high), urban (yes/no), income (low/medium/high), smoking (yes/no), alcohol drinking (yes/no), dietary intakes, other types of physical activity (total physical activity for sleep duration);

^c^Model 3: Further adjusted for hs-CRP.

^d^T, tertile.

### Joint association between physical activity, sleep duration and insulin resistance

**Figure 1:**
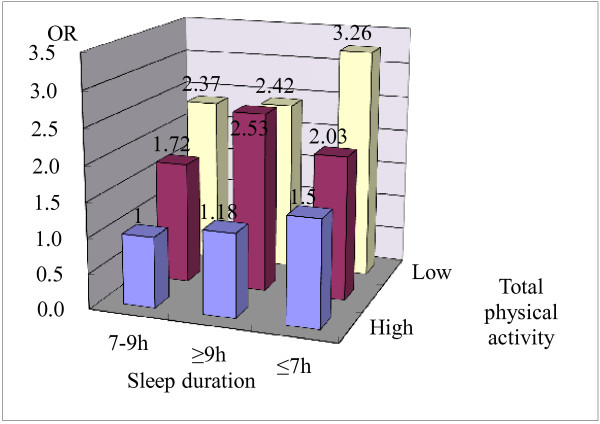
**Combined effect of total physical activity and sleep duration on insulin resistance by multiple logistic regression model adjusted for age, sex, WHR and income**.

We further assessed the combined effect of physical activity and sleep duration on insulin resistance in this study. As shown in Figure 1, lower total physical activity was related to higher odds of insulin resistance among different sleep duration categories. Short sleep duration was related to higher odds of insulin resistance in low and high total physical activity categories. In particular, the combination of low physical activity and short sleep duration had the highest odds of insulin resistance (adjusted OR = 3.26, 95% CI: 1.57-6.78), compared to the combination of high physical activity and appropriate sleep duration.

## Discussion

In this cross-sectional study, we found that total physical activity mainly composed of occupational activity, was strongly inversely associated with fasting insulin and insulin resistance. More importantly, we further assessed the independent association between different types of physical activity and HOMA for the first time in Chinese population. We identified a negative association between occupational physical activity and insulin resistance, which was found to be robust since results remained consistent throughout the analyses independent of potential confounders. In addition, transportation and domestic physical activities were also observed to be negatively associated with insulin resistance despite of relative weak association. There was a joint effect of low physical activity and short sleep in relation to insulin resistance.

In our study, non-exercise physical activity, especially occupation activity, contributed vast majority of total physical activity. Such physical activity pattern are similar in developing countries [10,36], but different from that in developed countries [37]. Therefore, it is necessary to analyze the independent association between individual components of physical activity and insulin resistance.

A number of studies have consistently demonstrated the inverse association between physical activity and insulin resistance [7,10,38,39]. Physical activity was independently associated with HOMA in both sexes among adolescents from Europe [38] and in US [7]. Physical activity could reduce insulin resistance and improve insulin sensitivity in adults [40,41]. Similar results were found in Iranian adults, together with the result that work-time physical activity had a higher contribution to such an association [10]. Our results not only confirm these previous findings, but also extend the four types of physical activity both separately and combined in relation to insulin resistance. A dose-response association was also detected in different types of physical activity.

Domestic physical activity and transportation activity was also shown beneficial effect for health [8-10,42]. Hu *et al. *[8] found that moderate and high physical activities including commuting physical activity independently and significantly reduced risk of Type 2 diabetes among the middle-aged general population. Again, he found that daily commuting to and from work reduced the risk for total and CVD mortality among patients with type 2 diabetes [9]. Esteghamati [10] reported a significant negative relationship between commuting activity and insulin resistance. Domestic physical activity was also observed having gender-specific effects on health indicators in Europe [42]. Negative associations between transportation/domestic physical activities and insulin resistance, despite of relative weak magnitude, were also observed in current data, which was consistent with what they found to a large degree.

Unexpectedly, a positive association was found between leisure physical activity and HOMA both in crude model and the model after adjustment for age and gender in our study. It can possibly be explained by that persons performing more leisure physical activity would also do less occupational physical activity. As we can see, in the higher quartiles of HOMA, where the leisure time physical activity was high, the occupational physical activity was lower. The mean occupational activity among those with leisure physical activities was significantly lower than those without leisure physical activities (50.1 vs. 132.1 MET-hrs/week, *p *< 0.001). Therefore, it is not the high leisure physical activity that is associated with the high HOMA but rather the high leisure time activity might be reflective of lower occupational activity which itself was associated with a high HOMA. Furthermore, people who were diagnosed with chronic diseases could have purposefully increased leisure time activity or exercise which could not be detected in our study due to cross-sectional nature. It has been shown that leisure physical activity is an effective method to reduce the risk of insulin resistance and impaired glucose tolerance [43], type 2 diabetes [8], obesity [42], the risk of metabolic syndrome, and all-cause mortality [44].

Physical activity is now recognized as a key component to lifestyle modification strategies. In US, the Centers for Disease Control and Prevention and the American College of Sports Medicine recommend that every US adult should accumulate 30 min or more of moderate-intensity physical activity on most, preferably all, days of the week [45]. More recently, the Federal government has issued the "2008 Physical Activity Guidelines for Americans" [46], which is more flexible for everyone to follow. The China Diet Guide (2007) issued by China Ministry of Health also recommends that an adult should have body activity equivalent to or more than accumulative 6000 steps every day. It is better to have moderate activity for 30 min if a person is in good shape [47].

To the extent that non-exercise physical activity (occupational, domestic and transportation) constituted a major source of physical activity, it is important to consider the synergistic effect by combining them together when developing relevant intervention strategies [48]. Particularly, it appears that occupational physical activity has a great potential to decrease insulin resistance and therefore to reduce the risk of adverse health outcomes, especially in developing countries like China.

Short sleep duration was related to impaired glucose tolerance, and type 2 diabetes [16-19]. It may be partially indicative of psychological stress, while the latter is also regarded as quasi-indicator of insulin resistance. We found a similar association in this study. Interestingly, a combination of low physical activity and short sleep duration had the highest odds of insulin resistance among all combinations, which had not been reported before.

The present study has several mentionable strengths. Firstly, the use of concept of metabolic equivalent (MET)-hours-per-week generated more precise estimation which takes both intensity and time spent on different types of activity into consideration. Second, different types of physical activity were assessed separately and independently. The associations found are more reliable since they were controlled for numerous confounders including diet, demographic factors, physiochemical parameters, and so on. Third, the questionnaires used in the study were developed with a standard method [29] and a Chinese language version was used.

The main limitation of our study is its cross-sectional nature, as was previously stated. Recall bias may exist in our study due to the use of questionnaires. Additionally, the "gold standard" for evaluating insulin resistance is the euglycemic hyperinsulinemic clamp method [49]. Due to its invasive nature, cost, we prefer to use HOMA instead in such a large epidemiological study. However, HOMA has been globally used as a reliable surrogate method in measuring insulin resistance [50-52].

## Conclusions

In summary, our findings indicate a significant inverse association between physical activity and insulin resistance in this non-diabetic Chinese population. Occupational physical activity, the main component of total physical activity, underlies this association. The combination of low physical activity and short sleep duration was associated with the highest odds of insulin resistance in the study. Advocating various types of physical activity and appropriate sleep duration may help reduce insulin resistance and its adverse consequences.

## Competing interests

The authors declare that they have no competing interests.

## Authors' contributions

HZ and ZS contributed to study design, conduct, data collection and statistical analysis, manuscript writing and revision. BY, YD and GW contributed to design, conduct and data collection. GH contributed to data analysis and manuscript revision. AH contributed to design, data analysis and manuscript revision. All authors read and approved the final manuscript.

## Pre-publication history

The pre-publication history for this paper can be accessed here:

http://www.biomedcentral.com/1471-2458/12/247/prepub
